# The Development of a Cost-Effective Imaging Device Based on Thermographic Technology

**DOI:** 10.3390/s23104582

**Published:** 2023-05-09

**Authors:** Ivo Stančić, Ana Kuzmanić Skelin, Josip Musić, Mojmil Cecić

**Affiliations:** Faculty of Electrical Engineering, Mechanical Engineering and Naval Architecture, University of Split, R. Boškovića 32, 21000 Split, Croatia; istancic@fesb.hr (I.S.); jmusic@fesb.hr (J.M.);

**Keywords:** thermal vision device, microcontroller, microbolometer, thermal imaging, histogram equalization, image enhancement

## Abstract

Thermal vision-based devices are nowadays used in a number of industries, ranging from the automotive industry, surveillance, navigation, fire detection, and rescue missions to precision agriculture. This work describes the development of a low-cost imaging device based on thermographic technology. The proposed device uses a miniature microbolometer module, a 32-bit ARM microcontroller, and a high-accuracy ambient temperature sensor. The developed device is capable of enhancing RAW high dynamic thermal readings obtained from the sensor using a computationally efficient image enhancement algorithm and presenting its visual result on the integrated OLED display. The choice of microcontroller, rather than the alternative System on Chip (SoC), offers almost instantaneous power uptime and extremely low power consumption while providing real-time imaging of an environment. The implemented image enhancement algorithm employs the modified histogram equalization, where the ambient temperature sensor helps the algorithm enhance both background objects near ambient temperature and foreground objects (humans, animals, and other heat sources) that actively emit heat. The proposed imaging device was evaluated on a number of environmental scenarios using standard no-reference image quality measures and comparisons against the existing state-of-the-art enhancement algorithms. Qualitative results obtained from the survey of 11 subjects are also provided. The quantitative evaluations show that, on average, images acquired by the developed camera provide better perception quality in 75% of tested cases. According to qualitative evaluations, images acquired by the developed camera provide better perception quality in 69% of tested cases. The obtained results verify the usability of the developed low-cost device for a range of applications where thermal imaging is needed.

## 1. Introduction

A sensor that can measure temperature is found today in many products, ranging from common household appliances to highly sophisticated machines. Devices that can sense and measure object temperature can be roughly divided into contact and non-contact ones [1]. Contact-type sensors (thermocouples, resistance thermometers, thermistors) are reliable, cost-effective, and simple but require direct contact with the sensing object to measure the temperature of that object. Contrary to contact sensors, non-contact temperature sensors can measure an object’s radiation energy from a distance, thus enabling many new applications [2]. Some non-contact sensors, such as pyrometers, bolometers, and photon detectors, are commonly used today. According to Planck’s law, terrestrial objects mainly emit in the LWIR (long wavelength infrared) band (7–14 μm), the range in which most microbolometers operate [3,4]. A focal plane array (FPA) is a special type/configuration of microbolometer and is used as a thermal sensor in infrared cameras.

Infrared images (IR) greatly depend on the thermal properties of the observed object and its surroundings. All objects above absolute zero temperature (−273.15 °C) emit infrared radiation. The infrared range is commonly divided into three regions: near-infrared, middle-infrared, and far-infrared [5]. At normal body temperature, humans radiate heat at wavelengths around 10 μm, which is part of the far-infrared spectrum. For this reason, the far-infrared region is also called thermal infrared (a term that will be used throughout this paper). Several types of thermal sensors are used today to acquire a thermal image, which differ in spectral response, response time, and sensitivity [2,3].

Lately, microbolometers have become the most popular thermal sensors due to their cost-effectiveness and portability. They do not require active cooling, have short start-up times, and can be obtained for less than EUR 200 per sensor module (when compared to more capable quantum detectors). Additionally, the disadvantages come with a low cost as microbolometers produce low sensitivity, noisy, and relatively small resolution thermal images [2,4] In theory, the cheapest and simplest microbolometer sensors with proper image enhancement can be used as an effective thermal imaging device, allowing object recognition in the scene (e.g., track path) while still easily detecting primary heat sources (e.g., other humans, animals, vehicles in motion). As compared to contemporary IIT (image intensifier tubes) devices or digital NIR (near infrared) cameras, thermal cameras benefit from the ability to see through smoke and mist [6]. Modern microbolometer modules have a high dynamic range which goes up to 14 bits [7]. Without any image enhancements, raw images exhibit contrasts that greatly exceed the range of modern display devices. While off-the-shelf display modules usually utilize 8-bit image levels description (i.e., 256 grayscale intensity levels), in some special medical applications, a 10-bit display may be utilized. However, Kimpe and Tuytschaever in [8] suggest that 10-bit resolution displays would be optimal for average human vision.

The level of incident IR radiation at the sensor depends on many factors and emissivity is one of the most important. Emissivity is defined as a property of a real object, characterizing its ability to radiate energy. The object’s emissivity can be defined as a ratio of excitance (emissive power) of the object’s surface and the theoretical emission of an ideal black body of the same size shape and temperature [9]. It is a unitless parameter with a value in the range between 0 and 1. This means that two different objects in the scene at the same temperature during the nighttime (e.g., tree bark and grass) can be perceived differently in thermal images. In real scenarios, those differences are small and visible only when a detailed analysis of the full spectrum is performed. Another important property of an object’s surface is reflectance, whereby the intensity of each pixel depends on both emitted and reflected radiation coming from nearby heat sources (e.g., atmosphere) [3,9].

Most commercial-grade thermal cameras employ some techniques for enhancing infrared images, which are directly implemented in the camera [10]. Techniques used in the enhancement of images greatly depend on the main purpose of the camera, e.g., radiometric cameras (cameras which can estimate the temperature of each spot in the image) minimally manipulate the originally acquired data. In contrast, cameras used for nighttime observation (e.g., hunting cameras and scopes) utilize a complex image enhancement algorithm, which aims to enhance details in the ambient while still keeping the main heat source (e.g., human or animal) recognizable. Thermal cameras equipped with the simplest automatic contrast control may result in losing important information that can be extracted from observed scenery. Such an example is a “blinding effect” [11], when a very hot object is present in the camera’s field of view. The camera will adapt its settings to the object’s temperature while making other important elements of the observed scene indistinguishable and the camera unusable in such scenarios. Thus, the question that motivated us emerges: is it possible to pair a low-cost microbolometer sensor with a low-power, off-the-shelf microcontroller running a customized image enhancement algorithm and an image displayed on a small display to achieve a useful and cost-effective night vision device?

The rest of the paper is organized as follows: in Section 2, related work is reviewed and contributions of the paper are outlined. Section 3 discusses hardware and the selection of hardware components. Next, implementation details are given in Section 4. Hardware supporting the algorithm is explicated in Section 5, while in Section 6, verification of the developed device through quantitative and qualitative evaluation is provided, followed by a discussion of the evaluation results in Section 7. The paper concludes with Section 8.

## 2. Related Work and Contributions

### 2.1. Applications and Hardware

Thermal cameras are increasingly popular in different consumer-oriented applications. One of the markets driving commercial thermal imagining is the automotive market, where thermal imagining is used for pedestrian and car detection for increased security and collision avoidance [12,13,14]. However, there are other applications such as object inspection in construction [15,16,17], medicine [18,19], veterinary science [20], access control [21], agriculture [22], astronomy [23,24], gas leakage detection [25,26], inspection of solar panels [27], autonomous drone navigation [28], helping visually impaired subjects [29], and so on. While a more in-depth presentation of thermal imaging applications can be found in [2,30]; the application range is evident and diverse.

In [13], a system for avoiding animal–vehicle collisions was developed. The system used a histogram of oriented gradients (HOG) algorithm for image enhancement and a convolutional neural network (CNN) for wild animal detection. It was tested with wild deer images captured with a FLIR One Pro thermal imaging device and achieved a detection accuracy of 91% [31]. Besides HOG, another histogram-based method used in thermal imaging applications is CENTRIST (CENsus TRansform hISTogram), which in [32] demonstrated a better performance in human detection in terms of accuracy and computational cost. Thermal images can also be used to improve autonomous vehicle safety during low visibility conditions [33]. Here, the authors fused thermal images (acquired with five FLIR Automotive Development Kit cameras with 20 fps) with radar data in a near real-time manner. In [12], the authors fused data from two spectrums—visible light and infrared light—to increase image readability. This was achieved on a field programmable gate array (FPGA) device with an image fusion competition approach based on image quality parameters. However, no results have been presented to demonstrate the effectiveness of the approach. A similar approach, based on fusing visible and thermal image data, was used in [34], where the authors used an encoder-decoder type neural network to fuse the two information channels. It is worth noting that large producers of thermal cameras, such as FLIR, encourage the fusion of visible and thermal cameras by providing large and well-organized databases for testing for free https://www.flir.eu/oem/adas/adas-dataset-form/ (accessed on 26 April 2023).

Combining thermal imaging with neural networks is also used in other works, as in [35], where it was applied for pedestrian detection. The authors used a custom thermal image database (recorded with FLIR ThermaCAM P10 thermal camera) and YOLO-based neural networks to detect pedestrians near the road’s edge. The obtained results showed that the YOLO network needed to be additionally trained to increase the average precision (AP) parameter up to 30%. At the same time, an identical network had a 90% AP on visible range images. Besides direct detection of objects of interest in thermal images, neural networks can be used to enhance them. This approach was used in a two-step manner in [31]. At first, the thermal image was translated to a grayscale image using Texture-Net, which was based on generative adversarial architecture. Then, the image was colorized using a deep neural convolutional network. In [36], several U-net-based neural network architectures were tested in infrared image coloring. The authors reported good results and noted that the approach achieved 3 fps without special optimization. Some works, such as [37], for medical-based applications are focused on algorithms for image segmentation and region of interest selection since this can have a significant influence on certain applications, such as medical thermography [38,39]. A good overview of state-of-the-art methods emphasizing available thermal image pedestrian databases can be found in [40]. A more technical overview of thermal image sensors can be found in [41].

It should be noted that one of the driving forces of thermal image application is thermal camera availability which can even be used as a smartphone add-on (as FLIR ONE, https://www.flir.com/flirone/) with good practical results [42]. Interestingly, a smartphone-based approach can be used for advanced applications with appropriate signal processing, as was the case in [19]. Here, a thermal image of the patient’s back was used to determine if the patient had COVID-19 disease. The obtained results showed 92% sensitivity and 0.85 area under the curve. While these and other commercial thermal cameras are becoming increasingly available and powerful, they are still too expensive for many targeted applications and are sold as black boxes. Thus, some works aim to develop affordable and open thermal imaging devices [43,44].

### 2.2. Image Enhancement

As was demonstrated above, once the thermal imaging process (i.e., data acquisition) has been completed, data processing algorithms come into focus since they can significantly influence overall system performance and overcome some of the limitations present in image acquisition systems (usually poor contrast, low resolution, lack of color information, etc.). These algorithms also contribute to the increased performance of subsequent image analysis algorithms (e.g., object detection). When working with thermal images, two main objectives should be considered: dynamic range reduction and detail enhancement [45] since a high-bit per pixel raw image needs to be mapped onto low 8-bit gray values (before false colorization). The two most widely used approaches are based on automatic gain control (AGC) and histogram equalization (HE). HE is the most notable representative of global contrast enhancement (GCE) methods in which one global mapping function is used (as opposed to local enhancement methods—LCE—where multiple local maps are used) [46]. Its main disadvantage is that grayscale values with a high probability distribution function will take out most of the intensity range and thus be significantly enhanced, while parts of the image with a low probability distribution function (foreground, which is usually of interest) will be suppressed. Thus, the approach over-enhances the homogeneous regions of the image and/or amplifies noise in the background. To avoid the issue, a number of improvements can be made to the original algorithm [47], with plateau histogram equalization (PHE) being a good example [48]. In the PHE, the probability density function is limited by a threshold. The idea can be evolved even further with a double plateau (i.e., two thresholds) [49]: an upper threshold to eliminate over-enhancement of background noise and a lower threshold to preserve details in the image. A short overview of HE algorithms can be found in [50]. Approaches also exist that try to leverage the advantages of both the local and the global-based approaches by fusing them [51].

LCE Histogram-based methods are widely-used contrast enhancement techniques since they are rather simple and intuitive both in theory and implementation [52]. One of the widely used algorithms in many thermal imaging applications is contrast limited adaptive histogram equalization (CLAHE) [53] alongside its variations (such as [54,55]) and fusions with other algorithms (as in [56]). It is based on adaptive histogram equalization (AHE) but avoids one of its pitfalls of over-amplifying noise in homogeneous image regions. It is part of the LCE group of enhancement methods since it divides the input image into multiple non-overlapping blocks and applies the algorithm to them. It uses interpolation to smooth out inconsistencies between borders of non-overlapping blocks. The algorithm has two parameters that can be tuned: the clip limit (that controls noise amplification) and the number of tiles (that controls the number of non-overlapping areas in the image). Lately, there have been attempts to improve CLAHE performance by estimating its parameters using supervised machine learning algorithms [52]. Other approaches to parameter optimization are also used, such as multi-objective meta-heuristics, which includes a structural similarity index (SSIM) [57] and entropy-based approach [58]. One way of tackling the issue is to use predefined histogram models, e.g., for an ideal far-infrared image consisting only of emissions and which would be a piece-wise constant [59]. Alternatively, non-parametric methods, such as the one in [60], can completely avoid the parameter selection procedure.

There are also approaches that separate raw images into two channels: base and detailed ones [45,56,61]. The base channel is usually processed for noise removal and/or compression, while the detailed channel is used to gain control during subsequent channel fusion. Fusion can also be implanted on per image bases as in [62] where a CLAHE processed image was used for the fusion of visible and infrared images using sparse representation. Another way of improving the CLAHE algorithms is to provide image/situation-specific information to the approach [63]. This motivated our approach where additional information was also used for image enhancement (temperature of the surroundings alongside a predefined histogram model). This approach, however, might be lacking for applications of long-range surveillance [59] due to possible large changes in the surrounding temperature. The CLAHE algorithm is also used in other image processing algorithms, not only for thermal images. It is also well suited for field programmable gate array (FPGA) real-time implementation [64]. It is worth noting that some authors (as in [65]) state that, in thermal images, GCE-based methods are better since they preserve thermal distribution information, which is important for some temperature-sensitive applications.

Based on the above review, we concluded that, due to a large number of possible application scenarios, there exists a need to develop a fully functional thermal imaging device based on off-the-shelf microcontrollers and appropriate (efficient) image enhancement algorithm(s). Thus, the contributions of the paper can be summarized as follows:

- Inclusion of ambient temperature information in the image enhancement algorithm to enable an increase in image contrast while preserving consistency with human visual perception, thus making it an effective night vision device;
- Adjustment of such an algorithm for execution on off-the-shelf microcontrollers, enabling full thermal module FPS while being comparable (in terms of performance) with commonly-used algorithms;
- Development of cost-effective, microcontroller-based, energy efficient (∼920 mW power consumption), completely functional device with a fast start-up time (∼300 ms). The device is based on readily available off-the-shelf components and is optimized for seamless real-time operation;
- Building of a small, fully-annotated dataset used in algorithm validation. The dataset includes the camera’s raw sensor data and ambient temperature, which can then be used to develop new image enhancement algorithms.

## 3. Materials and Methods

This project’s main objective was to develop a low-cost, battery-operated thermal monocular with low power requirements, fast start-up times, and the implementation of an advanced image processing algorithm that can provide an enhanced thermal image.

In the rest of this section, the discussion of hardware and selection of hardware components used for the proposed project will be elaborated, while the newly proposed thermal image enhancement algorithm will be described in detail and evaluated in the following sections.

### 3.1. Thermal Imaging Sensor

The most important component of the device is the thermal imaging sensor. A wide selection of thermal imaging sensors, known as camera cores, are available, and the designer must choose based on resolution, sensitivity, power consumption, complexity of integration, availability, and affordability.

Generally, uncooled thermal imaging technology based on microbolometer is the most common and provides basic functionalities without the requirement of an ITAR (International Traffic in Arms Regulations) license (https://www.federalregister.gov/documents/2020/01/23/2020-00574/international-traffic-in-arms-regulations-us-munitions-list-categories-i-ii-and-iii (accessed on 26 April 2023)). Their price ranges from EUR 200 for the simplest 80 × 60 FLIR Lepton module (https://www.flir.com/products/lepton (accessed on 26 April 2023)) and goes up by several thousand EUR for modules such as FLIR Boson operating at a resolution of 640 × 480 (https://www.flir.com/products/boson-plus/ (accessed on 26 April 2023)). More capable and costly camera cores are available today, which are not considered in our project due to the cost-effectiveness of the device.

FLIR Lepton^®^ is a complete, long-wave infrared (LWIR) camera module designed to interface easily with native mobile-device interfaces and other consumer electronics. It captures infrared radiation input in its nominal response wavelength band (from 8 to 14 microns) and outputs a uniform thermal image. Some models have an integrated radiometry option that provides calibrated temperature images. The introduction of the Lepton series of thermal modules into the market significantly impacted the availability and affordability of thermal imaging devices. Notable examples are the CAT S series of a smartphone (https://www.catphones.com/en-gb/cat-s62-pro-smartphone (accessed on 26 April 2023)) and FLIR ONE series of smartphone attachment thermal imaging cameras (https://www.flir.eu/flir-one (accessed on 26 April 2023)). Several other companies are offering smartphone-attached and ready-to-use out of box thermal camera modules, such as the InfiRay T2 module (https://www.infiray.com/products/thermal-camera-for-smartphone/ (accessed on 26 April 2023)) or Seekthermal Compact series (https://www.thermal.com/compact-series.html (accessed on 26 April 2023)). Another key advantage of the Lepton series to other similar camera cores is the availability of a breakout board (currently in version 2.0), which enables simple integration of camera cores to different projects and computer/microcontroller architectures (https://www.flir.com/products/lepton-breakout-board-v2.0 (accessed on 26 April 2023)). As several Lepton modules are available (Lepton 1.5, Lepton 1.6, Lepton 2, Lepton 2.5, Lepton 2.6, Lepton 3, and Lepton 3.5), we have chosen the currently most capable Lepton 3.5 module with on-board thermal radiometry, 160 × 120 resolution and thermal sensitivity less than 50 mK. The integrated and non-removable silicone lens offers a horizontal field of view of 57°. Due to export requirements, the camera frame rate is fixed at 8.7 Hz (<9 Hz), which is common in this range of devices. The stated power requirements in the datasheet are 160 mW during normal operation and 800 mW during a shutter event for a short period (less than 1 s) when NUC (non-uniformity correction) is performed, which has to be considered while designing the power supply.

Lepton utilizes SPI (serial peripheral interface) for video interface and two-wire I2C (inter-integrated circuit) serial as a control interface, which enables implementation on common microcontroller boards. The Lepton 3.5 module was placed into a FLIR Lepton® Breakout Board (version 1.4) with a smaller footprint than the latest version 2.0 and only eight pins for interfacing.

### 3.2. Microcontroller

**Figure 1:**
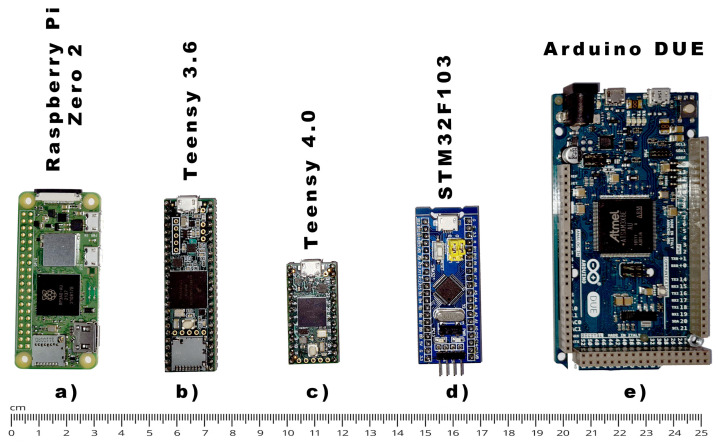
Microcontroller boards and computer boards evaluated during the development of the proposed device; (**a**) Raspberry Pi Zero 2, (**b**) Teensy 3.6, (**c**) Teensy 4.0, (**d**) STM32F103, (**e**) Arduino DUE.

The second component that had to be carefully selected as the embedded computer board in charge of image acquisition from the thermal module, advanced image processing and, finally, image representation on the OLED display. We have considered and successfully tested several computer boards; one notable example is the Raspberry Pi Zero 2 (https://www.raspberrypi.com/products/raspberry-pi-zero-2-w/ (accessed on 26 April 2023)), shown in Figure 1a), for which, despite its high performance (1 GHz quad-core processor and 512 MB RAM), slow boot-up times made it suboptimal for our project (due to the use of MicroSD as primary non-erasable memory). In general, microcontrollers are much smaller in footprint and are targeted for specific applications. They have a simpler microprocessor than computer boards (e.g., Raspberry pi) along with memory (RAM and ROM) and some peripherals, all embedded in a single chip. Microcontrollers are generally limited to the memory size (both ROM and RAM) and the selection of interfaces (WiFi, Bluetooth, Ethernet, etc.). We have considered commercially available microcontroller boards only, which contain a microcontroller, I/O pins, power regulators, protection circuitry, and an interface for easy programming (most commonly a USB interface). Today, a range of microcontroller boards are available. However, we considered only ARM-based high-performance boards with the maximum available amount of RAM. Over the last decade, the emergence of ARM Cortex cores in micro-controller products (https://www.arm.com/products/silicon-ip-cpu (accessed on 26 April 2023)) has diminished the challenges for embedded developers. Most high-performance microcontroller suppliers now have an ARM processor, which excludes the popular 8-bit AVR cores used in the Arduino family, making the ARM Cortex core the current industry standard.

Currently, the Teensy 4.0 microcontroller board (https://www.pjrc.com/store/teensy40.html (accessed on 26 April 2023)), shown in Figure 1c), is the fastest off-the-shelf microcontroller that can be used out of the box for complex calculations and high-bandwidth interfacing. It features an ARM Cortex-M7 processor (please note that most alternative boards host ARM Cortex-M3 or M4) with an NXP iMXRT1062 chip clocked up to 600 MHz. The Teensy 4.0 microcontroller features 1024K RAM (of which 512 K is tightly coupled) available for storing local data, which is of the most importance since all image enhancement algorithms require several instances of the whole image to be stored in computer RAM. During the development stage, several other boards were considered, such as STM32F103C8T6, known as the “blue pill” (Figure 1d), and Arduino Due (Figure 1e), which were both based on the Arm Cortex M3. Unfortunately, the low amount of available RAM (20 KB and 96 KB) makes them virtually useless for any complex image processing application, as compared to Teensy 4.0 1024 KB of RAM, which allows storing several complete images inside SRAM. Additionally, both boards have a larger footprint than Teensy 4.0, as can be seen in (Figure 1). During the initial development phase, a setup with the older generation Teensy 3.6 was used (Figure 1b), as it has an integrated MicroSD card slot which was required for storing acquired images, but was in later stages replaced with a more capable Teensy 4.0 alternative.

### 3.3. Display

The third component that has to be carefully selected is the display compatible with the microcontroller’s SPI or I2C interface. Today, there are only two technologies available on the market, namely LCD (liquid crystal display) displays or OLED (organic light emitting diode) displays, while E-paper displays are not considered due to high latency and the fact that they are mostly monochromatic. An OLED display works without a backlight where the emissive electroluminescent layer is a film of organic compounds that emits light. From a usability point of view, it can display deeper black levels than LCD and can be thinner and lighter than LCD of comparable size. That means that when a user uses the device in the complete dark, the OLED display can provide a readable image without compromising the user’s night vision due to strong backlight. We have selected Freetronics OLED128 (https://www.freetronics.com.au/products/128x128-pixel-oled-module (accessed on 26 April 2023)) as an optimal solution, with contains an active display area of 28.8 × 26.8 mm with 128 × 128 resolution in full-color RGB. Small modifications of the provided library (https://github.com/freetronics/FTOLED (accessed on 26 April 2023)) enable us to tweak/reduce the operating voltage, thus providing a non-blinding and readable nighttime image. Freetronics OLED128 utilizes the SPI interface and has been successfully tested up to a 20 MHz clock rate. The provided library also enables developers to create simple objects on display (lines, circles, characters, etc.) or to create a custom image in a pixel-per-pixel fashion without requiring to operate directly with the displays controller with low-level commands. The OLED display cannot be used as an eyepiece out of the box, as any monocular would require one. For this reason, a simple housing was designed for holding the OLED and 1${}^{\prime \prime }$ focusing eyepiece lens.

### 3.4. Other Components

As our algorithm requires high-precision ambient temperature measurement (description follows in the next sections), we considered several temperature sensors. The TMP117 (https://www.ti.com/product/TMP117 (accessed on 26 April 2023)) was selected, as it provides a 16-bit temperature reading with a resolution of 0.0078 °C and an accuracy of up to ±0.1 °C. The sensor utilizes an I2C interface and can be conveniently integrated within a selected microcontroller board.

**Figure 2:**
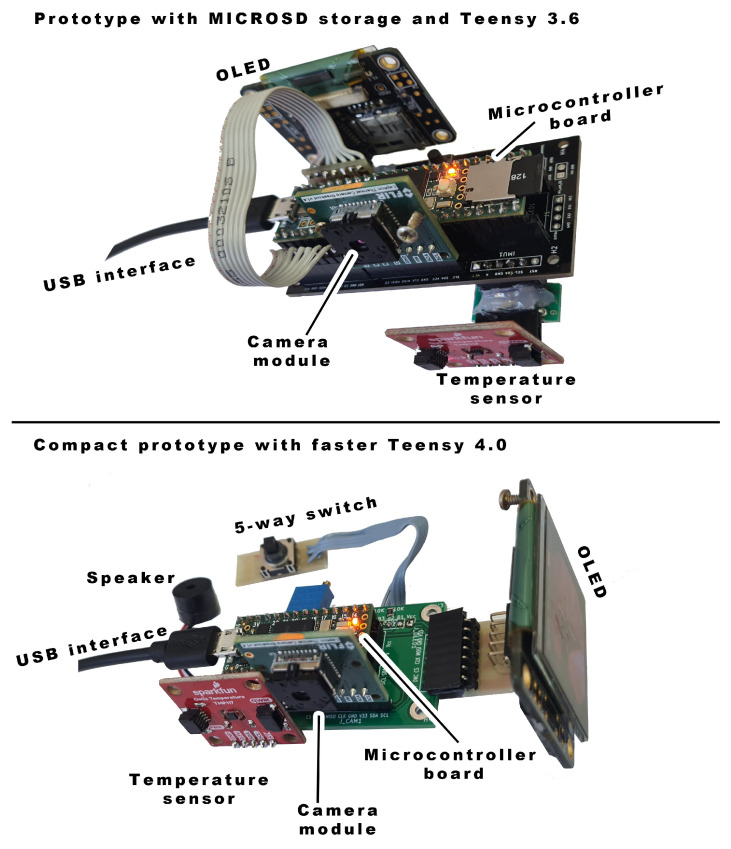
Two prototypes were used during development with marked key components: Teensy 3.6 based with storage capabilities used for dataset acquisition (**top**) and more compact and faster Teensy 4.0 based for algorithm testing (**bottom**).

The initial configuration with a Teensy 3.6 microcontroller and in-house developed PCB is depicted in Figure 2. As mentioned before, this version was used only for testing the hardware capabilities and acquiring image samples which were used for developing an image enhancement algorithm.

**Figure 3:**
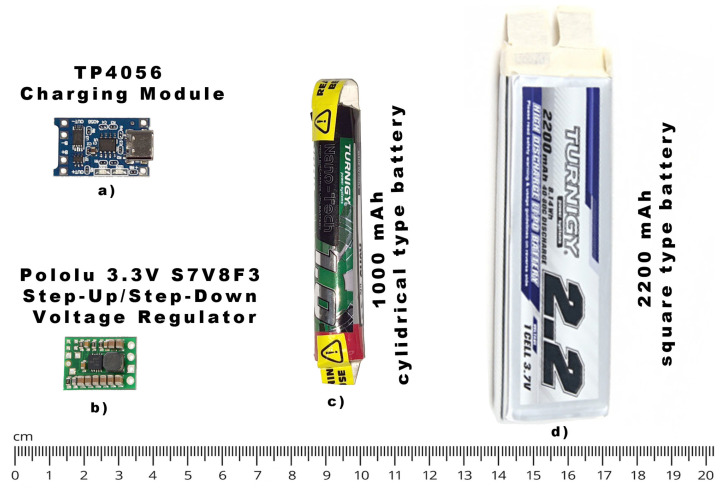
Size comparison of power supply components used during the development of the proposed device: (**a**) charging module, (**b**) voltage regulator, (**c**) smaller 1 Ah battery, (**d**) larger 2.2 Ah battery.

Finally, we considered a few battery and voltage regulators to enable autonomous battery operation. We selected a Turnigy single-cell LiPoly square battery of 2200 mAh capacity and with cell size 97 × 34 × 9 mm, as shown in Figure 3. Please note that almost any battery of acceptable shape and capacity may be sufficient if the battery can be fitted inside the housing, such as Tunergy cylindrical LiPoly battery shown in Figure 3b. A slim and square-like shape simplified the housing design by enabling us to place the battery in the base of a monocular. A Pololu 3.3V S7V8F3 Step-Up/Step-Down Voltage Regulator was in charge of providing stable voltage output towards the microcontroller and other peripheral modules (https://www.pololu.com/product/2122 (accessed on 26 April 2023)). This regulator proved to be the most reliable as it can provide up to 1 A (500 mA continuous) even when the input voltage is near-minimum (2.7 V), which normally occurs when the battery is almost depleted. The TP4056 Charging Module enabled battery charging with a USB-C connector. The TP4056 is a constant-current/constant-voltage linear charger ideally suited for many portable applications holding single-cell lithium-ion batteries. Additionally, the module contains battery over-discharge protection and battery overcurrent protection as our battery cell does not contain additional protection electronics. The measured current consumption for the complete device was on average 280 mA at 3.3 V (920 mW), which enables up to 7 h of battery autonomy.

Some additional sensors were also considered and partially implemented in the test configuration but were omitted in the final version due to our intention to create the simplest possible design; namely, BMP280 atmospherics sensors for measuring humidity and air pressure (https://www.bosch-sensortec.com/products/environmental-sensors/pressure-sensors/bmp280/ (accessed on 26 April 2023)), a Bosch BNO055 orientation sensor for drawing artificial horizon on display (https://www.bosch-sensortec.com/products/smart-sensors/bno055/ (accessed on 26 April 2023)), and an additional Melexis MLX90614 contactless temperature sensor for additional calibration (https://www.melexis.com/en/product/mlx90614/digital-plug-play-infrared-thermometer-to-can (accessed on 26 April 2023)). All mentioned sensors are I2C compatible and could be easily integrated using a shared I2C interface (which TMP117 and camera core already use). Atmospheric data may improve the accuracy of thermal reading and compensate for attenuation of thermal radiation due to air between the object and camera, while in the current version of our algorithm, we focused primarily on enhancing the thermal image.

**Figure 4:**
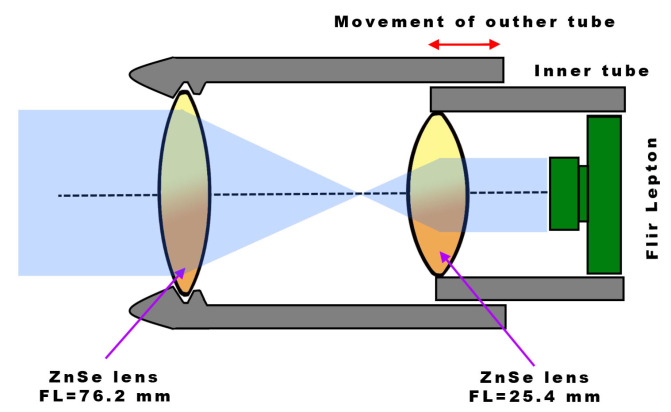
Simple Keplerian system-based lens assembly which enables 3× magnification and manual focusing.

As the original Lepton’s horizontal FOV of 57° is too wide for some applications, an additional lens configuration was considered to enable a narrower FOV and thus increase the detection/recognition range. As the simplest solution, an additional lens configuration following the Keplerian system was selected, as it requires only a pair of of-the-shelf ZnSe (Zinc Selenide) convex lenses, commonly used as a focusing lens for a CO${}_{2}$ engraving laser. As depicted in Figure 4, the lens closer to the sensor was 20 mm in diameter and 25.4 mm in focal length, while 25 mm in diameter and 76.2 mm focal length with anti-reflective coating was used as a second lens, thus effectively creating a 3× magnification (calculated 20.5° horizontal FOV).

### 3.5. Housing and PCB

**Figure 5:**
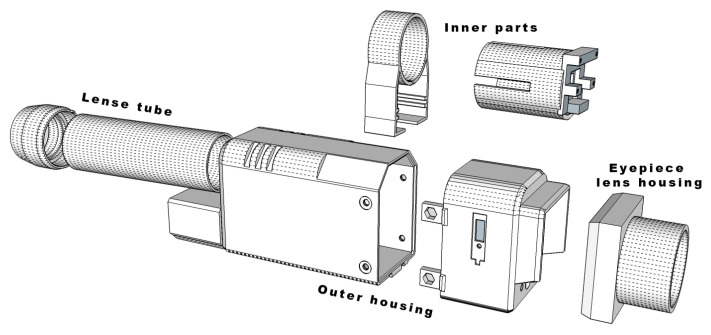
Sketch of 3D printed components which acts as device housing and holder of the internal components.

All components were placed inside in-house-designed and 3D-printed housing, as seen in Figure 5. The housing was divided into components: the core component that holds the camera module, OLED display, battery, and microcontroller, the lens assembly that holds ZnSe lenses, and the outer shell that holds all components together. The outer lens tube allows slight extrusion in order to adjust optimal focusing for indented distance. An integrated five-way micro switch was used as input from the user. Together with two status LEDs, a power switch, an ambient temperature sensor, and connectors were blended inside the outer shell. As the main printing material, we used tough PLA (https://ultimaker.com/materials/tough-pla (accessed on 26 April 2023)) while printing was carried out using an Ultimaker2+ 3D printer with a 0.4 mm nozzle (https://ultimaker.com/3d-printers/ultimaker-2-plus-connect (accessed on 26 April 2023)).

**Figure 6:**
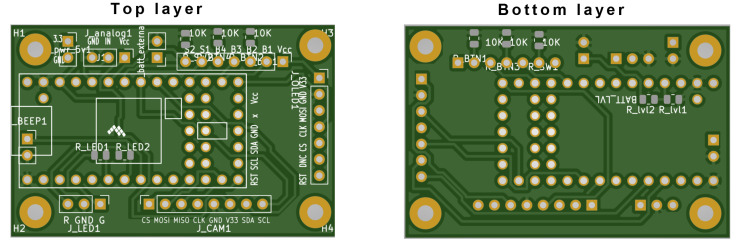
Visualization of all layers of the Printed Circuit Board for the compact Teensy 4.0 based prototype, the **top** and **bottom** layer.

Double layer PCB (printed circuit board) with dimensions of 50 × 33 mm was designed, which holds the microcontroller and interface to other electronic components, see Figure 6. Additionally, a voltage divider was implemented onboard for measuring battery voltage. The PCB had four mounting holes on the edges to secure it to the core 3D-printed component.

**Figure 7:**
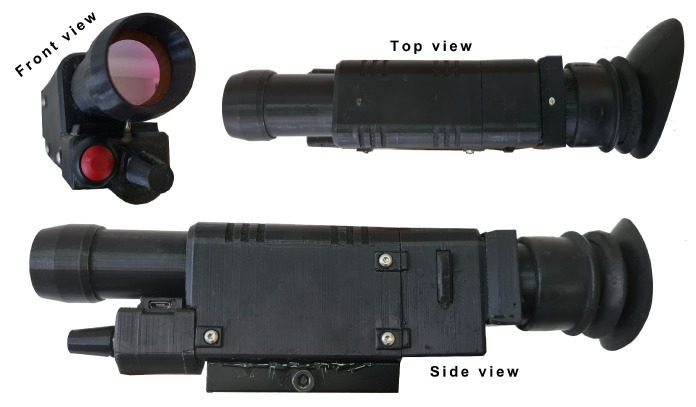
**Top**, **bottom**, and side view of the completed imaging device.

The fully functional final product with all integrated components is presented in Figure 7. Parts of the housing were tightened with steel screws, while port openings (to programming USB interface and charging port) were additionally protected with rubber covers. The eyepiece part contained an additionally mounted rubber eyepiece protector, which prevented ambient light from entering the observer’s eye during nighttime use.

A list of all critical components used in the prototype, together with exact model names and cost (acquired in April 2023), is given in Table 1. Several components, such as the microbolometer breakout board, temperature sensor, and voltage converter, use independent and costly modules (breakout boards), which could all be integrated into a main PCB to reduce total prototype cost and volume.

## 4. Implementation

The completed system should offer a smooth user experience without the noticeable delay of an image on display or sudden freezing due to unexpected calculations. The single microcontroller is responsible for all image acquisition operations, reading data from sensors and buttons, performing advanced image enhancement, and delivering image frames to a display. The camera core is the “slowest” component, due to legal/export reasons, fixed to an 8.7 Hz frame rate which led us to our goal to design a whole cycle that is limited to 115 ms. This was achieved by utilizing pre-calculated lookup tables (LUT) in most of the code instead of directly calculating results for each instance. When recalculation of LUT was required in frames, other time-intensive operations such as checking for new sensor data, were skipped. In practice, ambient temperature and battery voltage do not change instantly. Hence, the timing of any advanced operation was easy to schedule and balance. We investigated the maximum useful SPI clock rate for both the camera core and display, thus minimizing the time required for transferring images from the camera core to the microcontroller and from the microcontroller to the display. The SPI library used with the Teensy microcontroller allowed the instant change of SPI parameters for each device using SPI setting and SPI transactions. FLIT Lepton 3.5 showed stability with a clock frequency of up to 24 MHz, while the OLED clock rate had to be reduced to 20 MHz. Faster clock rates were possible but may occasionally result in instability and short freezing of the system due to how Lepton handles transmission errors (de-asserting the SPI line for at least 185 ms).

**Figure 8:**
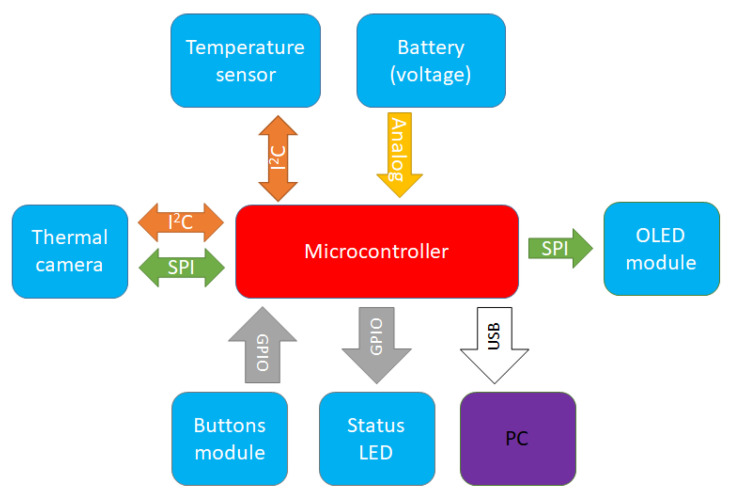
Hardware interfaces utilized on the proposed device. Each interface between the component and microcontroller/computer is colored differently.

As SPI is the fastest interface available on modern microcontroller boards, both the display and Lepton module rely on them for fast data transfer from and to the microcontroller, see Figure 8. The Lepton module utilizes an additional I2C (Inter-Integrated Circuit) as a control interface, used only during camera initialization in the setup stage, after which the Lepton module continuously streams image data when its SPI chip selection line is asserted. The ambient temperature sensor is the only module that continuously utilizes the I2C interface after the initialization stage, whereas the other I2C compatible sensors (as mentioned in Section 3) could be easily implemented to utilize the same shared I2C interface without significant modification on hardware or software. A battery voltage sensor is a simple resistor voltage divider using a single microcontroller’s analog-enabled pin and internal ADC periphery to calculate correct battery voltage levels. Status LEDs and user input via a buttons module or a 5-way switch use any free GPIO pins. If required, simple debugging (status logs and calculated parameters) was managed over the 480 Mbit/s USB interface toward the computer (PC).

The Lepton uses an SPI-based VoSPI protocol (https://www.flir.eu/developer/lepton-integration/lepton-tech-docs/ (accessed on 26 April 2023)), which does not require timing signals. The VoSPI segment is defined as a continuous sequence of VoSPI packets consisting of one-quarter of a frame (80 × 60 pixels) of pixel data. Each VoSPI packet contains a single video line of data where each pixel required 2 bytes of data (164 bytes, 4 bytes header, and 160 bytes payload) when the default RAW14 video format was used. The code for handling the Lepton VoSPI stream was written from scratch and optimized for the best microcontroller performance, including acquiring thermal images and handling possible errors in transmission.

Although the TFOLED library allows the creation of complex graphical objects to be displayed on the screen, this library primarily aims for relatively underpowered microcontrollers. As the Teensy 4.0 microcontroller holds 1MB of RAM, the whole image, including all additional graphical elements, was prepared directly in the microcontroller’s memory and then transferred as a whole frame to the OLED, thus minimizing transfer time and, most importantly, delays (flickering) when OLED is updating new graphical objects.

**Figure 9:**
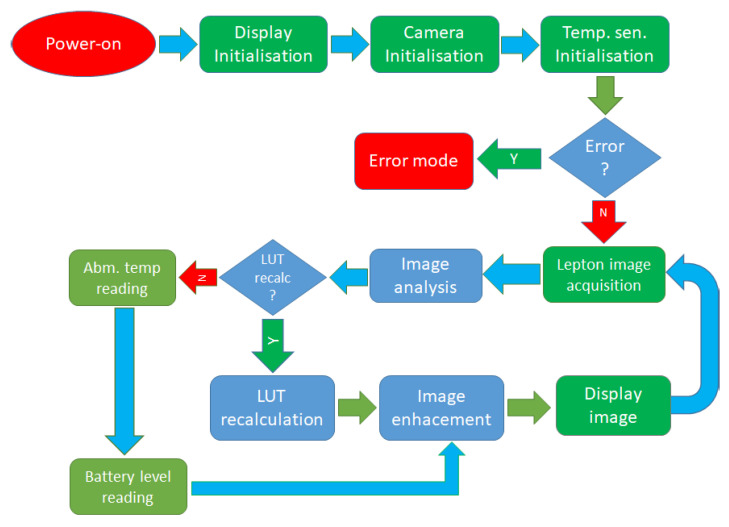
Flowchart presenting order of operations of the proposed device.

The flowchart presented in Figure 9 describes the orders of operations of the proposed device. When powering on, the device goes through the initialization stage, where all connected modules are properly initialized and checked for errors. If any error is detected, the device goes to a permanent error state where a user is informed by a status-led flashing and beeping sound. On the contrary, devices transfer the thermal image to the microcontroller when no errors are detected and perform image analysis. If the statistical parameters of the image are drastically changed, it requires LUT recalculation, which is promptly executed. If parameter changes are insignificant, the device continues to sensor reading (temperature and battery level). The operation is followed by advanced image enhancement procedures, which are explained in detail in the following sections. In the last step, an image with additional graphical elements is prepared and transferred to an OLED display, after which the loop continues indefinitely with Lepton image acquisition.

## 5. Proposed Algorithm

**Figure 10:**
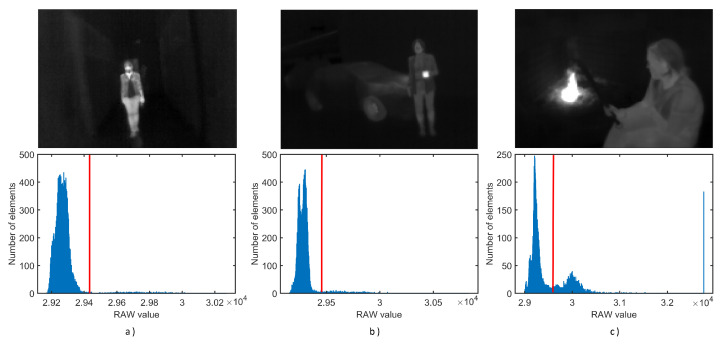
Example of a histogram (**bottom**) and the corresponding unedited thermogram (**top**) for three selected scenes—(**a**) Person in the corridor; (**b**) Person holding a hot beverage in the parking garage near a car; (**c**) Person sitting near a fireplace. Ambient temperature in histograms is marked with a red line.

The proposed algorithm allows a noticeable improvement of the thermal image quality and therefore increases the effectiveness of both ambient visibility and the detection of warm-blooded subjects. Implementing such an algorithm on hardware limited microcontroller is a challenge dealt with in this paper. As mentioned in the related work section, few thermal image datasets are available for developing our image enhancement algorithm or improving an existing one. To the best of our knowledge, none of them include ambient temperature recording with high precision. Adding a high-precision temperature sensor to the setup helps us to determine the threshold which contributes to the segmentation of the image into regions that belong to heat-producing objects (such as warm-blooded animals, vehicles, or machinery) or regions that are part of the passive ambient, i.e., background. The emphasis was given to increasing the contrast of background objects, where the intensity of received radiation differs insignificantly for objects at the same ambient temperature. Note that this algorithm was primarily derived for nighttime purposes, where levels of reflected thermal radiations were minimal. Thus, only emitted radiation is received by sensors, where objects, if imaged, are at the same temperature and differ only in small variations of emissivity coefficients due to different materials and surface finishing (e.g., dirt, tree bark, or vegetation) or the state of the same materials (e.g., wetness of dirt). Measuring the correct ambient temperature with a high-precision temperature sensor can help us extract pixels (objects) that emit heat with temperatures much higher than ambient temperature (e.g., warm-blooded animals and fire). With this approach, the algorithm could separate high-temperature pixels, which in the original “raw image” histogram unnecessarily occupy numerous levels and do not contribute to the readability. In contrast, pixels below the threshold usually occupy a much smaller portion of levels (Figure 10) and are essential for the final enhanced image readability.

The radiometry-enabled mode changes the pixel output to represent the scene in Kelvin temperature values, where each level corresponds to a 0.01 K difference. For example, a pixel value of 30,000 signifies that the pixel is measuring 26.85 °C (300.00 K − 273.15 K). This should be adequate, as the stated thermal sensitivity is <0.05 K or five levels of intensity, which means that even small variations of radiations can be observed.

In the proposed algorithm, the image was statistically analyzed upon acquisition, and a histogram was created for all possible levels (16,384 levels) of temperature, after which two new histograms were derived: one representing background objects and another one representing active heat-emitting objects, see Figure 10. The threshold is determined by the maximum temperature of background objects if their emissivities are close to 0.98, which is a relatively high coefficient for common materials and surfaces, while emissivity coefficients are in practice much lower and would report lower temperatures than the ambient temperature [66].

The specificity of thermal image histograms, as compared to grayscale image histograms taken with a common CCD (charge-coupled device) or CMOS (complementary metal-oxide-semiconductor) camera, is that the thermal image histogram is sparsely populated and normally exists in only a few tenths of possible levels (as shown in Figure 10, the only section of full scale is populated). Thus, image enhancement techniques commonly utilized on RGB or grayscale images do not result in satisfactory results. The histogram presented in Figure 10c) is an example of where a local high-temperature source (fire) causes overpopulation of the last histogram bin due to the limitation of maximum temperature that the device can correctly measure and interpret.

The proposed algorithm can be generalized to any displaying device and its properties concerning available color modes and usable grayscale intensity levels. The final displayed grayscale image should contain a preselected number of levels (e.g., 128) where one section is reserved for background “ambient” pixels (e.g., 1–96) and the rest for foreground “warm” pixels (e.g., 97–128), a ratio which is user-selectable and does not affect algorithm performance at all.

**Figure 11:**
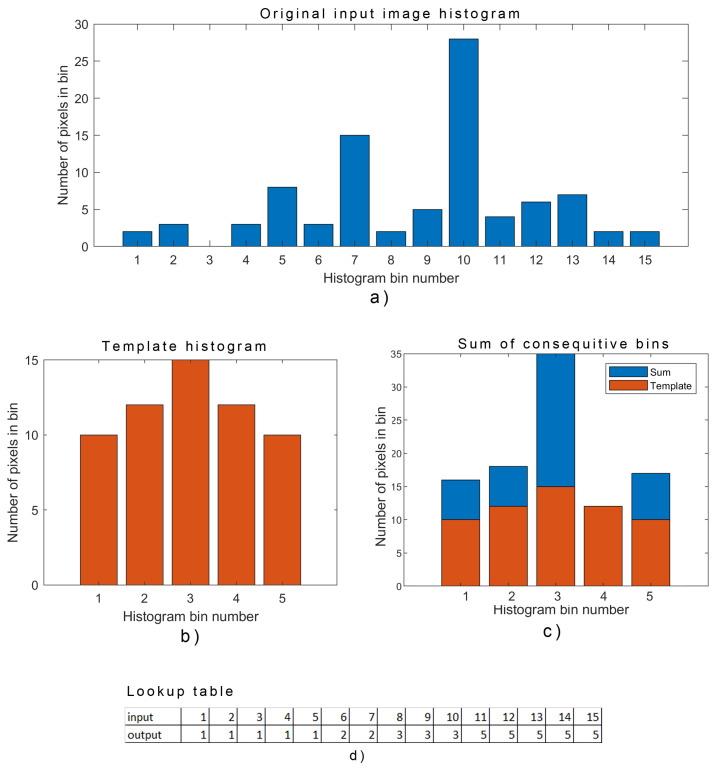
Mapping of input image levels to final lookup table. (**a**) Input image histogram; (**b**) Template histogram; (**c**) Sum of consecutive bins from original image histogram; (**d**) Lookup table.

**Figure 12:**
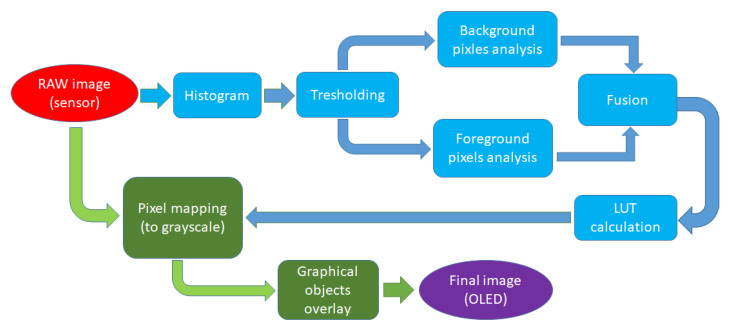
Flowchart describing image enhancement algorithm implementation on the micro-controller.

The steps of our proposed algorithm are explained in detail as follows:

- The empty template lookup table is created, which contains the same number of elements as the input image has levels (e.g., 16,384 for 14-bit image);
- A histogram for the whole input image is calculated to determine the number of pixels below $numpix_{low}$ and over the threshold $numpix_{high}$, Figure 11a);
- A template histogram is created with a predefined number of bins (e.g., *i* = 128) where each bin size $maxbinsize_{i}$ is calculated based on the number of pixels below threshold $numpix_{low}$ and number of bins (i) and where the template histogram follows the Gaussian distribution, Figure 11b);
- An input image histogram (with 16,384 levels) is analyzed bin per bin where several pixels in consecutive bins are summed until they reach $maxbinsize_{i}$. Values in a template lookup table (for summed consecutive bins) are updated and hold the number of the targeted bin, Figure 11c);
- If the sum of consecutive bins greatly exceeds $maxbinsize_{i}$, a few consecutive values in the template lookup table may be left empty (with value 0). The number of “skipped” values $n_{skip}$ in the lookup table depends on overpopulation size and is calculated as follows:
  $$n_{skip}=\log_{2}\frac{sum_{pixels}}{maxbinsize_{i}}.$$
- After all bins of the input image histogram are analyzed, a single lookup table is created, which contains pixel mapping for all values below the threshold to a new smaller number of values. Nevertheless, the targeted number of values was predetermined (e.g., 128) actual number may vary due to the uncertain number of pixels in input histogram bins and skipped values in the lookup table, Figure 11d);
- An additional lookup table (final lookup table) is then created with a fixed number of elements (e.g., 96) where all values from the template lookup table are linearly mapped to a new smaller final lookup table;
- A similar procedure is executed for all histogram levels of the input image over the threshold, where the final lookup table is updated accordingly, (e.g., values from 97 to 128), Figure 12.

Parameter $maxbinsize_{i}$ for each bin from the template histogram is defined by the number of pixels below or over the threshold and a predefined number of bins, which can be tweaked by the user and adjusted according to the utilized display properties. Suppose several bin parameters are set to low. In that case, it will result in suboptimal use of the display (e.g., not all 256 levels will be used), and the much higher number would produce an enhanced image that the embedded display cannot effectively reproduce (and would require OLED with higher grayscale bit depth). In our setup, we used a fixed number of bins set to 128 for all test images, which results in, in most cases, a dynamic range of provided display being optimally utilized. The result of this part of the algorithm is a large lookup table (LUT) in which, for each raw pixel level, there exists a single targeted pixel level for the final grayscale image, see Figure 12. Each new thermal image is statistically analyzed and, if distributions do not differ significantly, recalculations of new LUT are skipped for this cycle. In this case, the final grayscale image is created as a simple pixel mapping which is extremely fast on the microcontroller. We purposely indicate grayscale images, as only grayscale images can be objectively compared with common evaluation methods. Modifications to a basic histogram equalization algorithm drastically increase the contrast in image segments for objects with similar thermal properties while still maintaining the contrast for objects whose thermal properties significantly differ (e.g., sky, water surfaces, vegetation). This two-step histogram equalization allows us to distinguish foreground objects from the background and keep high contrast levels, thus enabling subsequent identification of the targeted object while optimally utilizing available and very limited displays.

Additionally, graphical elements such as crosshair, battery voltage levels, and ambient temperature were overlaid on the original image and are displayed.

## 6. Results

**Figure 13:**
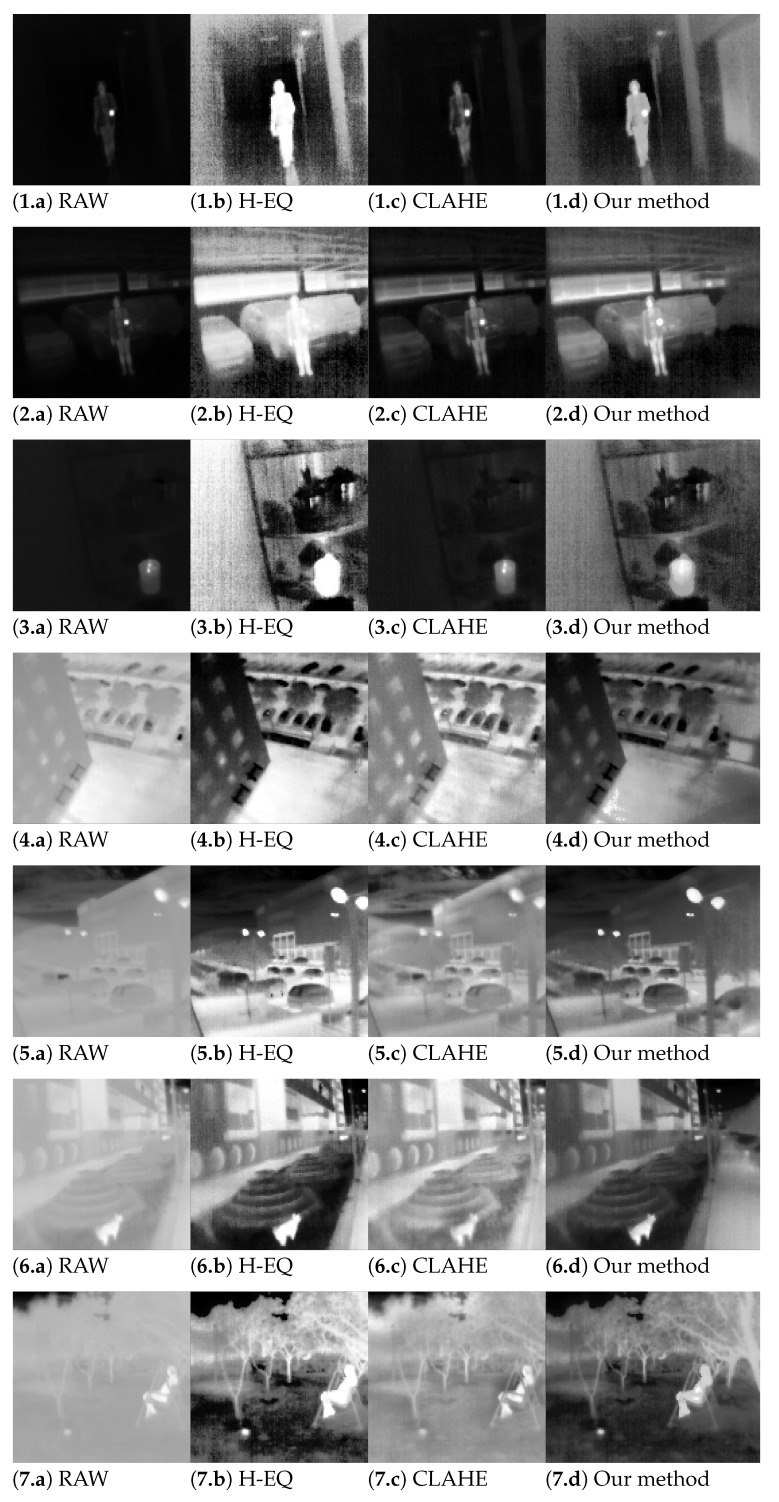

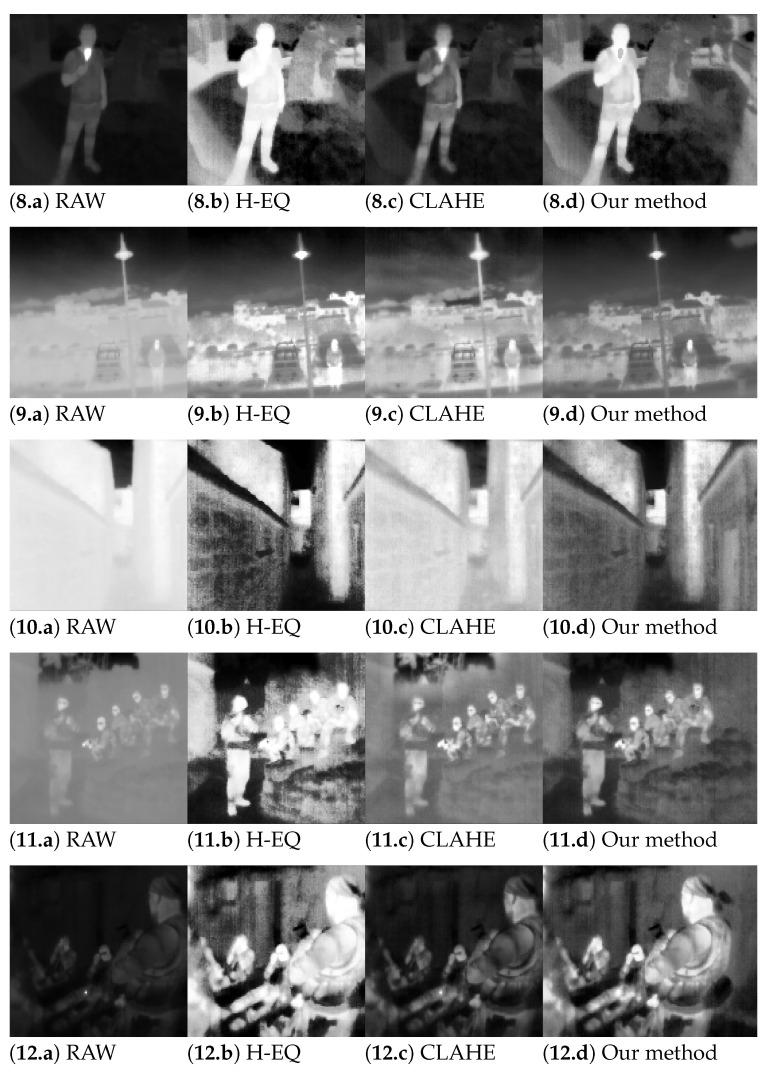
Raw images (**1.a**–**12.a**) and results of image enhancement; History equalization (**1.b**–**12.b**); CLAHE (**1.c**–**12.c**); our method (**1.d**–**12.d**).

This section presents detailed performance results using several different metrics which illustrate the effectiveness of the proposed algorithm both in an objective and subjective manner. The results were compared with several well-known (standard) algorithms from the field. A set of 12 thermographic images from a new database (out of 74 thermographic images in total) were selected for testing. These images were selected semi-randomly to choose random images but to cover all 12 scene types in the database. These scene types were agreed upon and recorded before testing (i.e., they were not tailored to suit our new algorithm) and were recorded to include both the nighttime and daytime situations of people near strong thermal sources (such as an open fire or a hot cup of tea), scene with and without people including different types of scenarios (e.g., urban and marine), etc. These twelve images are presented in the first column of Figure 13.

### 6.1. Analysis of the Execution Time

As mentioned in previous sections, the targeted system FPS (frames per second) was set to 8.7 (maximum allowed fps of the camera core) and recalculated in terms of milliseconds, one cycle lasting exactly 115 ms. This time is available for reading images from the camera core, enhancing the image, performing additional operations with the microcontroller, and finally transferring the enhanced image to the OLED for displaying. Firstly, we measured the time required for the microcontroller to transfer the image to the OLED, which was constant and equaled 19 ms. Other operations executed by the microcontroller were simple and required less than 1 ms in total. These included reading states from the 5-way button, battery voltage measurement, etc., and due to their small time footprint they were omitted from further analysis. It was not a straightforward task to calculate the time required for transporting the image from the camera core to the microcontroller, as the camera core waits until the next image frame is available, thus effectively prolonging the measured read time. On the other hand, if the image enhancement algorithm should perform a more complex calculation and the image frame is not completely read before the next image frame is available, the camera core loses synchronization with the microcontroller and requires resynchronization which requires approximately 185 ms. This occurrence was observed as a short freezing of the device. By forcing an additional delay time in the main loop, we calculated the maximum time available for the image enhancement algorithm (before experiencing resynchronization), which was approximately 18 ms, thus allowing an extra 5 ms for our proposed algorithm. Our algorithm proved to be highly efficient on targeted microcontrollers and requires 13 ms to execute on average. For reference, we also tested execution times for simple histogram equalization (2.5 ms), linear normalization (less than 1 ms), and CLAHE (21 ms), where only CLAHE implementation suffered from constant resynchronization and experienced FPS reduction to 4.5. Other more complex techniques [2,10] require high computing power paired with the sensor, which makes them impractical on a small scale and cost-ineffective.

**Figure 14:**
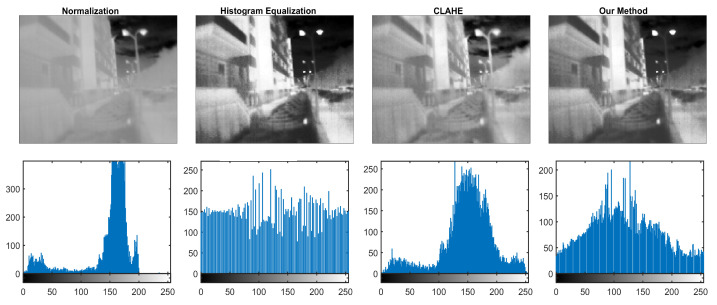
Example of output grayscale image **(top row**) and corresponding histograms (**bottom row**).

For insight into how each method performs, Figure 14 illustrates the processed images and corresponding histograms for all associated methods. The normalization method is closest to the original sensor reading as it only implements min-max normalization into a 256-intensity value. Illustrating the unedited original reading in full 16-bit depth would appear as a primarily uniform and practically unusable image.

### 6.2. Quantitative Evaluation

To objectively evaluate the performance of the proposed system, three no-reference quality assessment metrics were adopted: blind/referenceless image spatial quality evaluator (BRISQUE), [67], lightness order error (LOE), [68], and entropy (ENT) [69]. The BRISQUE is a metric providing statistics of pair-wise products of neighboring luminance values, as defined in [67]. The lower BRISQUE score indicates better image quality. The LOE provides the lightness order error between the input image and its enhanced counterpart. A lower LOE score indicates better image quality. The ENT measures the richness of details and contrasts through information content calculations. The higher ENT score indicates an image with richer details. The quantitative evaluation results of the three methods in terms of the three metrics are given in Table 2, Table 3 and Table 4, respectively. Please note that the RAW image set refers to the image obtained from the image sensor and does not include any additional processing. Additionally, no RAW set was included in Table 3 due to how the LOE parameter was calculated, i.e., it compares the processed image with the original raw one.

From the presented results, it can be seen that the proposed method performs best for all metrics: CLAHE in five out of twelve cases tied with H-EQ with the same result, LOE in eleven out of twelve cases, and ENT metric in eleven out of twelve cases. The proposed method was best in 27 out of 36 evaluation cases (75%). The runner-up was the H-EQ algorithm which performed best in six out of thirty-six cases (17%).

### 6.3. Qualitative Evaluation

The raw infrared images and experimental results for several different image enhancement algorithms are given in Figure 13. The experimental results were obtained by applying histogram equalization (H-EQ), contrast-limited adaptive histogram equalization (CLAHE) with an 8 × 8 block size and 0.02 clip limit, and the proposed method to the raw infrared images. Images with qualitatively better results were considered to provide better local contrast and noise suppression, without artifacts, and are visually pleasing.

**Figure 15:**
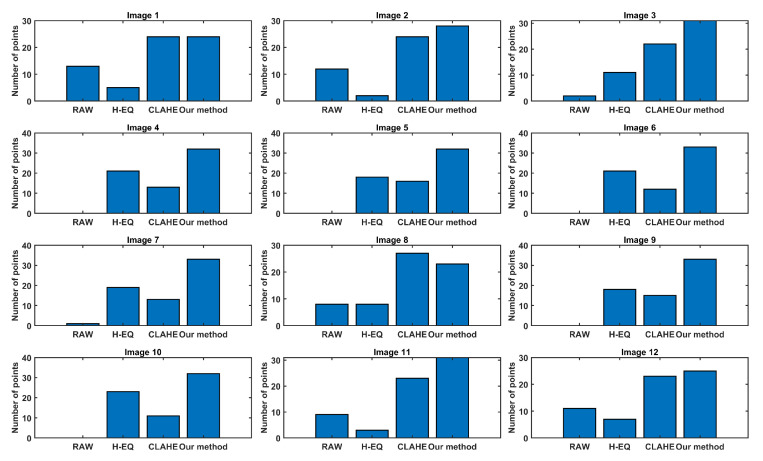
Results of the subjective evaluation based on 12 sample images for our algorithm (from **left** to **right** in each subfigure): RAW image, H-EQ image, CLAHE image, and image based on the proposed method.

To evaluate the resulting images more subjectively, the simple but effective test was performed on 11 test subjects (three females and eight males) who were not involved in developing the device. Subjects were presented with four images of the same scene on a big screen, one containing normalized RAW images and the other three containing enhanced images using different methods. Presented images were reordered by permutation in each scene. They contained only simple identification markings in the form of A, B, C, and D, where markings were kept in the same order. In this way, subjects were unaware of which enhancement method was presented with each image. The order of the presented scene was also permutated for each subject. Subjects were tasked with evaluating the quality of each method (in their own subjective view) by selecting the best, second best, and third best ones for each scene. The best representation was awarded three points, the second one two points, and the third one with one point. The summarized results are shown as a bar graph in Figure 15, where each method was evaluated for each scene, thus allowing us to analyze how our proposed method performs in different scenarios and setups. The proposed method obtained the best score for 10 out of 12 presented scenes. For one scene (Scene 1), the result is a tie with the CLAHE method, and in one scene (Scene 8), CLAHE is the best method according to the subjects’ votes. Those findings support our hypothesis that the proposed method should create visually the best images.

## 7. Discussion and Future Work

The main goal of this paper was to develop an imaging device supported by a highly optimized and low-resource requirement algorithm that enhances RAW thermal camera images for optimal visualization and shows details that were not previously visible on the RAW images. Twelve unique scene categories were selected from a larger dataset for evaluation and comparison. The evaluation was performed in two steps: a quantitative analysis by calculating BRISQUE, LOE, and ENT metrics. Surprisingly, by BRISQUE metrics, our algorithm ties with a simple H-EQ algorithm (both were best on five scenes), which led us to conclude that another quantitative method was required. Our algorithm performed best in 11 out of 12 scenes by LOE and ENT metrics, whereas an H-EQ or CLAHE approach showed better metrics in different scenes. The performed quantitative analysis was insufficient to evaluate the proposed algorithm, and an additional qualitative analysis had to be performed on the same dataset, which was carried out in the second phase. As the whole system was intended to enhance RAW images for better visualization while being observed primarily by different subjects, the qualitative analysis described in the previous section was shown to be inevitable. A more detailed analysis of the result, provided in Figure 15, showed that the proposed image enhancement algorithm provides the best results, especially in outdoor scenarios (examples include Scenes 3, 4, 9, and 11). In indoor scenarios (Scenes 1, 2, and 12), our approach was qualitatively evaluated by subjects as being similar to or slightly better than CLAHE. Interestingly, in a few scenes (Scenes 4, 5, 6, 9, and 10), CLAHE is not the runner-up, and simple H-EQ was evaluated by subjects to be a better solution. In a single scene (Scene 8), which contained a highly complex outdoor scenario, CLAHE was evaluated as the best solution and better than our proposed algorithm. As discussed before, neither CLAHE nor H-EQ offer good overall (general case) solutions, which depend heavily on the scene and this supports our effort to develop a new algorithm for thermal image enhancement. As can be observed from the results provided by both quantitative and qualitative evaluation, our proposal was not evaluated as definitely the best in all possible scenarios but was shown to be the best overall algorithm that can fulfill most of the real-life scenarios. As an additional comment to the presented results, we may note that the main aim was not to develop a competing algorithm but the computationally efficient supporting software for the imaging device. The quantitative evaluations show that, on average, images acquired by the developed camera provide better perception quality in 75% of tested cases. According to qualitative evaluations, images acquired by the developed camera provide better perception quality in 69% of tested cases.

As microcontroller-compatible color OLEDs can commonly offer limited grayscale levels (ranging from 32 to 128), their full visualization capabilities are unused when only presenting a grayscale image. Implementing advanced false coloring with our existing proposed method should help us better differentiate foreground objects and small details in the background and further increase visual perception. The basic idea is to use the HSI color model, where the method proposed in this paper controls intensity levels, where a new algorithm should be proposed for optimally controlling hue and saturation channels. This approach should involve a larger scale evaluation on a large pool of subjects, thus was omitted from this part of the research.

## 8. Conclusions

The research presented in this study aimed to explore the feasibility of creating a microcontroller-based thermal camera/monocular, which can be effectively used as a night vision device. The goal set was to provide instant boot-up time, low power consumption, and smooth real-time operation. As microcontroller processing power is limited, an optimized algorithm for thermal image contrast enhancement was proposed and presented in the paper. An additional evaluation of several algorithms commonly used today in similar devices proved that our proposed algorithm is best suited for its intended application, with numerical results comparable to or better than commonly used algorithms. The numerical analysis was further supported by subjective analysis of the resulting images when compared to the images resulting from other methods.

The experiment on hardware demonstrated that the proposed algorithm can be executed on a microcontroller in real-time, where the whole process of reading data from the sensor, advanced image enhancement, and representation of the enhanced image on an OLED display takes no more than 115 ms, thus utilizing the maximum available frame rate of the camera core (8.7 fps).

## Figures and Tables

**Table 1:** List of critical components used in the prototype, model names, and price in USD.

Component	Model	Cost in USD
Microbolometer sensor	Lepton 3.5	160 USD
Microbolometer breakout board	Lepton Breakout Board v1.4	40 USD
Temperature sensor	Sparkfun TMP117 module	13 USD
Microcontroller board	Teensy 4.0	23 USD
Display	Freetronics OLED128	28 USD
Battery	Turnigy 2200 mAh Lipo Single Cell	3 USD
Voltage converter	Polulu Voltage Regulator S7V8F3	11 USD
Other components	Buttons, LED, Charging connector, TP4056 battery charger	10 USD
Lense	ZnSe Lense 25 mm/76.2 mm FL, 20 mm /25.4 mm FL	60 USD
PCB	PCB manufacturing cost (CNC, edge cuts, drilling, mask)	2 USD
3D print material	Ultimaker tough PLA, 120 g	8 USD
Total cost		358 USD

**Table 2:** The quantitative evaluation results of histogram equalization (H-EQ), contrast-limited adaptive histogram equalization (CLAHE), and the proposed method in terms of BRISQUE (a lower score indicates better image quality). Best method for each sample is bolded in the table.

Metric: BRISQUE				
**Image No.**	**Raw**	**Method**		
		**H-EQ**	**CLAHE**	**Our Method**
1	41.05	32.75	40.94	**31.26**
2	37.58	**24.73**	34.97	26.41
3	34.83	34.02	36.75	**27.94**
4	28.92	34.35	37.00	**21.53**
5	31.82	**15.34**	39.03	20.40
6	33.92	**17.92**	35.15	21.25
7	31.63	33.59	32.95	**29.40**
8	43.03	43.08	43.13	**40.07**
9	35.25	**21.38**	31.58	22.64
10	44.50	**34.84**	41.68	35.17
11	**27.79**	34.35	34.94	36.52
12	**25.41**	41.36	34.73	32.64

**Table 3:** The quantitative evaluation results of histogram equalization (H-EQ), contrast-limited adaptive histogram equalization (CLAHE), and the proposed method in terms of LOE (a lower score indicates better image quality). Best method for each sample is bolded in the table.

Metric: LOE			
**Image No.**	**Method**		
	**H-EQ**	**CLAHE**	**Our Method**
1	125.67	364.51	**114.20**
2	114.75	191.12	**48.02**
3	**197.71**	1209.52	199.06
4	50.04	1061.59	**22.95**
5	109.51	1026.48	**38.27**
6	56.15	916.60	**28.33**
7	73.40	881.58	**52.98**
8	97.89	372.33	**38.88**
9	143.17	749.29	**44.56**
10	101.55	681.54	**76.92**
11	149.49	886.18	**76.39**
12	129.63	395.55	**39.33**

**Table 4:** The quantitative evaluation results of histogram equalization (H-EQ), contrast-limited adaptive histogram equalization (CLAHE), and the proposed method in terms of ENT (a higher score indicates the image with richer details). Best method for each sample is bolded in the table.

Metric: ENT				
**Image No.**	**Raw**	**Method**		
		**H-EQ**	**CLAHE**	**Our Method**
1	4.09	5.88	4.71	**6.63**
2	5.03	5.97	5.93	**7.31**
3	3.77	5.88	4.42	**6.36**
4	6.63	5.99	7.36	**7.50**
5	6.17	5.99	6.87	**7.56**
6	6.56	5.99	7.22	**7.51**
7	5.44	5.98	6.50	**7.47**
8	5.64	5.99	6.51	**7.72**
9	7.06	6.00	**7.69**	7.57
10	5.14	5.97	6.33	**7.07**
11	5.37	5.92	6.37	**7.28**
12	5.39	5.98	6.43	**7.64**

## Data Availability

The data presented and analyzed in, this study are openly available in: https://github.com/istancic/thermal_dataset (accessed on 26 April 2023).

## Abbreviations

The following abbreviations are used in this manuscript:

OLED	Organic Light-Emitting Diode
SoC	System on Chip
LWIR	Long Wavelength InfRared
FPA	Focal Plane Array
IR	Infrared
IIT	Image Intensifier Tubes)
NIR	Near Infra-Red
HOG	Histogram of Oriented Gradients
CNN	Convolutional Neural Network
CENTRIST	CENsus TRansform hISTogram
FPGA	Field Programmable Gate Array
AGC	Automatic Gain Control
GCE	Global Contrast Enhancement
LCE	Local Enhancement Methods
PHE	Plateau Histogram Equalization
CLAHE	Contrast Limited Adaptive Histogram Equalization
SSIM	Structural Similarity Index
ITAR	International Traffic in Arms Regulations
LWIR	Long-Wave InfraRed
NUC	Non-Uniformity Correction
SPI	Serial Peripheral Interface
PCB	Printed Circuit Board
LUT	Lookup Tables
I2C	Inter-Integrated Circuit
CCD	Charge-coupled device
CMOS	Complementary Metal-Oxide-Semiconductor
FPS	Frames Per Second
BRISQUE	Blind/Referenceless Image Spatial Quality Evaluator
LOE	Lightness Order Error
ENT	Entropy
H-EQ	Histogram Equalization
